# Listeria Meningitis Complicating Alemtuzumab Treatment in Multiple Sclerosis—Report of Two Cases

**DOI:** 10.3390/ijms160714669

**Published:** 2015-06-29

**Authors:** Daniela Rau, Michael Lang, Andreas Harth, Markus Naumann, Frank Weber, Hayrettin Tumani, Antonios Bayas

**Affiliations:** 1 Department of Neurology, University of Ulm, Ulm 89081, Germany; E-Mails: rau.daniela@gmx.de (D.R.); Hayrettin.Tumani@rku.de (H.T.); 2 Neuropoint Patient Academy, Neurological Practice Center, Ulm 89073, Germany; E-Mail: lang@neurologie-ulm.de; 3 Department of Neurology, Military Hospital, Ulm 89081, Germany; E-Mails: AndreasHarth@bundeswehr.org (A.H.); frankweber@bundeswehr.org (F.W.); 4 Department of Neurology, Klinikum Augsburg, Augsburg 86156, Germany; E-Mail: Markus.naumann@klinikum-augsburg.de

**Keywords:** alemtuzumab, multiple sclerosis, Listeria monocytogenes, meningitis

## Abstract

Alemtuzumab, a humanized monoclonal antibody targeting the surface molecule CD52, leads to a rapid depletion of immune cells in the innate and adaptive immune system. In phase 2 and 3 trials in multiple sclerosis (MS), infections have been reported more frequently in alemtuzumab than in interferon beta treated patients. Here we report two patients treated with alemtuzumab for MS developing Listeria meningitis few days after the first infusion cycle. Both patients recovered completely after prompt diagnosis and adequate treatment. Physicians and patients should be aware of this serious, but treatable complication.

## 1. Introduction

Alemtuzumab is a humanized monoclonal antibody, which targets the surface molecule CD52 on immune cells (T- and B-cells, monocytes, dendritic cells and thymocytes) and leads to a rapid and significant depletion of those cell types. In animal studies, lymphocyte numbers in primary and secondary lymphoid organs were maintained [1].

In 2013, Alemtuzumab (Lemtrada^®^) was approved for relapsing multiple sclerosis (MS) by the EMA, and by the FDA in 2014.

Well-known side effects include secondary autoimmune reactions mainly consisting of thyroid disorders, immune thrombocytopenia, anti-glomerular basement membrane disease and membranous glomerulonephritis, which may occur years after the first treatment.

Infections were more common with alemtuzumab 12 mg than with subcutaneous interferon beta-1a (sc IFNB-1a) in clinical phase 2 and 3 trials (CAMMS223: 66% *vs.* 47%; CARE-MS I: 67% *vs.* 45%; CARE-MS II: 77% *vs.* 66%) with most of them being mild or moderate in severity [2,3,4]. Serious infections were slightly more frequent in alemtuzumab *versus* sc IFNB-1a treated patients (CAMMS223: 3% *vs.* 2%; CARE-MS I: 2% *vs.* 1%; CARE-MS II: 4% *vs.* 1%), most commonly affecting the respiratory and urinary tract.

Opportunistic infections in alemtuzumab treated patients have been reported in single cases with MS and leukemia [5,6]. In MS, Listeria meningitis has been reported in a 36-year-old female receiving two annual cycles of alemtuzumab 24 mg/day, with symptoms starting 13 days after the last infusion [7].

Here we report two cases of Listeria meningitis occurring immediately after the first cycle of alemtuzumab infusions.

## 2. Case Reports

### 2.1. Case 1

A 47-year-old Caucasian female developed first MS symptoms in 1992. In the subsequent 10 years, the patient developed four relapses with optic neuritis and transverse myelitis, neuromyelitis optica was ruled out. Each relapse was treated with high-dose glucocorticosteroids resulting in an incomplete recovery. In 2002, immunmodulatory therapy with glatiramer acetate was started. Yet, new relapses occurred, prompting several treatment changes, furthermore, repetitive cycles of plasma exchange were necessary (see Table 1). Methotrexate had to be stopped in May 2013 because of persisting disease activity. In January 2014, alemtuzumab 12 mg daily i.v. over 5 days was started. The concomitant medication was applied as recommended in the SmPC.

**Table 1:** History of MS treatment and disease activity in case 1.

Time Period	Treatment/*n* = Relapses Under Treatment
2002–2006	Glatiramer acetate/*n* = 4
2006–2010	Natalizumab, plasma exchange/*n* = 6
2010–2011	Fingolimod, plasma exchange/*n* = 2
2011–2013	Methotrexate, plasma exchange/*n* = 2
2014	Alemtuzumab

The day following the fifth alemtuzumab infusion, the patient developed subfebrile temperatures and progressive cephalgia. On the third day, she reported fever up to 40.1 °C (104 °F), cephalgia, neck stiffness, photophobia and a generalized worsening of preexisting MS symptoms and was admitted to hospital. C-reactive protein (CRP) was elevated with 42.4 mg/dL (normal range: <0.8 mg/dL). Cerebrospinal fluid (CSF) analysis revealed a pleocytosis of 459 leukocytes/µL, predominantly neutrophils, CSF protein was elevated and lactate increased. In cultures of CSF (but not in blood), Listeria monocytogenes could be detected (Table 2).

An empiric treatment with ampicillin, ceftriaxone and aciclovir was initiated. After detection of Listeria monocytogenes, treatment was continued with ampicillin monotherapy for 21 days. Cranial MRI showed two new contrast-enhancing lesions, but no signs of Listeria encephalitis.

After starting antibiotic treatment, the patient’s condition improved rapidly with only mild cephalgia persisting for 2 weeks. The follow-up CSF examination 17 days after the diagnosis of Listeria meningitis revealed a mild pleocytosis (20 leukocytes/µL) with normalized lactate and negative CSF cultures (Table 2). At discharge, 21 days after admission, the patient had no sequelae. The patient denied any changes in food intake or intake of potentially Listeria-contaminated animal or herbal food.

**Table 2:** Clinical symptoms and CSF findings in case 1.

Days After Last Alemtuzumab Infusion	Symptoms	Findings	Treatment
d1	Subfebrile temperatures, progressive cephalgia	-	-
d3	Fever (40.1 °C, 104 °F), cephalgia, neck stiffness, photophobia, worsening of preexisiting MS symptoms	CSF analysis: Cell count: 459 leukocytes/µL (predominantly neutrophils) Protein: 0.966 g/L (normal range 0.080–0.45 g/L) Lactate: 7.4 mmol/L (normal range 1.2–2.1) Intrathecal IgM synthesis CSF cultures: Listeria monocytogenes positive Cranial MRI: 2 new contrast-enhancing lesions	After positive cultures for Listeria monocytogenes in CSF: ampicillin for 21 days
d17	Free of complaints	CSF analysis: Cell count: 20 leukocytes/µL Protein: 0.444 g/L Lactate: 2.14 mmol/L	Ampicillin continued

### 2.2. Case 2

First MS clinical signs in the 43-year-old female Caucasian patient occurred in February 2014 with symptoms caused by a transverse myelitis with sensory disturbances ascending to the chest, bladder dysfunction and a progressive deterioration of gait with loss of the ability to stand without assistance. Repeated glucocorticosteroid pulse therapies, finally with 2 g methylprednisolone, followed by immune adsorption resulted in a relevant, however incomplete recovery. In May 2014, the second relapse occurred, again a marked, but incomplete recovery under glucocorticosteroids could be observed with increasing sensory symptoms at the end of June 2014. Due to the high disease activity, a treatment with alemtuzumab was initiated with 12 mg i.v. daily over 5 days. Over the 5 days, the patient was treated concomitantly with methylprednisolone 1 g daily, for 3 days, as recommended by the SmPC, for two further days to improve tolerability of alemtuzumab and for reducing disease activity. Treatment was well tolerated apart from an exanthema starting on the 5th day of alemtuzumab infusion. Glucocorticosteroids were planned to be tapered off over one week.

Three days after the last alemtuzumab infusion, the patient developed subfebrile temperatures on the fourth day, with a fever up to 40.4 °C (104.7 °F). Antibiotic treatment with cefuroxim was initiated by the primary care physician. Since the patient’s condition did not improve and the CRP increased, the patient was admitted to the hospital. At admission, CRP was 16.7 mg/dL (normal range 0–0.5), WCC 11.87/nL with 97.3% neutrophils and 0.6% lymphocytes. Urine analysis as well as a CT-scan of the chest and abdominal ultrasound did not reveal an underlying cause. Empirically, an anti-infective treatment with gancyclovir and piperacillin/tazobactam was initiated. Two days after admission, 8 days after the last alemtuzumab infusion, the patient developed headache and meningism. The CSF revealed a pleocytosis of 195 leukocytes/µL (see Table 3). After lumbar puncture, the patient was immediately treated with ampicillin, ceftriaxone and gentamycin. Treatment with ceftriaxone was stopped after obtaining the culture results with detection of Listeria monocytogenes in blood and CSF cultures. Ampicillin and gentamycine were given for 3 weeks. The patient’s condition improved rapidly and she finally recovered completely. CRP normalized 9 days after the first lumbar puncture, and the follow-up CSF examination is shown in Table 3. CSF cultures were negative. After discharge, the patient was treated with trimethoprim/cotrimoxazole for one week (fourth week after diagnosis of meningitis) for prevention of a recurrence of meningitis.

**Table 3:** Clinical symptoms and CSF findings in case 2.

Days After Last Alemtuzumab Infusion	Symptoms	Findings	Treatment
d4	Fever up to 40.4 °C (104.7 °F)	-	-
d6	-	CRP 16.7 mg/dL (normal range 0–0.5), WCC 11.87/nL (97.3% neutrophils, 0.6% lymphocytes)	Cefuroxim, gancyclovir, piperacillin/tazobac-tam
d8	Headache and meningism	CSF analysis: Cell count: 195 leukocytes/µL (72% neutrophils, 23% lymphocytes, 5% monocytes/macrophages) Protein: 0.43 g/L (normal range 0.15–0.45 g/L) Lactate: 4.4 mmol/L (normal range 1.1–2.4 mmol/L) CSF and blood cultures: Listeria monocytogenes	Ampicillin and gentamycine for 21 days, followed by trimethoprim/cotrimoxazole for 7 days
d25	Free of complaints	CSF analysis: Cell count: 8 leukocytes/µL (2% neutrophils, 62% lymphocytes, 36% monocytes/macrophages) Protein: 0.18 g/L Lactate: 1.6 mmol/L CSF cultures: negative	-

There was no proven ingestion of raw milk products or contaminated food. However, approximately half a year before, reports about Listeria-contaminated grating cheese, packed in plastic bags, were published in the media that may have been ingested by the patient.

## 3. Discussion

After the first report of Listeria meningitis in the CAMMS223 trial, we present two additional cases in alemtuzumab-treated MS patients. Since both infections occurred briefly after the first infusions, immunosuppression induced by alemtuzumab has to be assumed as causative.

Listeria monocytogenes is a gram positive, facultative intracellular bacterium with the ability, after a variable incubation period from a few days up to 3 weeks, to induce gastroenteritis, but also meningitis, encephalitis, brain abscesses and rhombencephalitis in humans. Listeria infection, though it occurs rarely in humans (0.1 to 10 cases/million; 0.1% of all foodborne infections), is considered the most severe bacterial foodborne infection (among others, present in cheese, raw milk, strawberries, water, and smoked salmon) [8]. The lethality is up to 30% in case of neurological involvement, even if appropriately treated. Around 47% of the cases correspond to CNS-infections [9]. Listeria monocytogenes is mentioned as the second to fourth cause of community-acquired acute bacterial meningitis in adults, with known predisposing factors like immunosuppression, age over 50 years and underlying conditions such as diabetes or malignancy [8].

In Case 1, meningism developed two days after onset of subfebrile temperature, whereas in Case 2, five days after fever onset caused by nascent Listeria sepsis. Listeria monocytogenes-induced meningitis mostly has a subacute course. In 43% of Listeria monocytogenes meningitis cases, the classical triad of fever, neck stiffness and change in mental status has been reported [10].

Keeping in mind that typical signs of meninigitis may be absent in the beginning is important, since Listeria meningitis has to be considered in immunosuppressed patients with septicaemia without abnormal findings in neurological examination.

CSF findings typical for bacterial meningitis may be absent in up to 23% of patients (pleocytosis with neutrophil predominance in 77% of cases) and gram staining of the CSF is positive only in one third of cases, making culture of blood and CSF or a PCR-analysis necessary to detect the intracellular bacterium with a moderate sensitivity and specificity of 80% [10,11,12]. In both patients reported, CSF findings were consistent with Listeria meningitis, proven by blood (Case 1) and CSF (both cases) cultures.

After inoculation and crossing of the intestinal barrier, the bacterium is transferred via the lymphatic system and blood to its primary target organs, the liver (up to 80%–90%) and the spleen [13,14]. The innate immune system exerts the early immune response and control of the bacteremia within the first 6 h [14]. In that phase, Listeria monocytogenes is also rapidly internalized into host cells (*i.e.*, inflammatory monocytes, neutrophils, and hepatocytes) and it may spread by a cell-to-cell route [13,14]. Because of the intracellular lifecycle, bacterial clearance is now entirely dependent on secondary activation of cytolytic CD8 T-cells [15]. If the infection cannot be controlled by an adequate immune response in the liver and spleen, an unlimited proliferation of Listeria monocytogenes may result in the parenchyma with subsequent release of bacteria into the circulation invading other organs, especially the CNS [8,12,14]. In the case of an intact immune system, Listeria monocytogenes infection is generally cleared within 5–10 days (in mice and humans), but there is some evidence that in infected animals, low numbers of bacteria can persist in the gall bladder and bone marrow as an important reservoir of this pathogen, also with regard to a secondary infection of the CNS [15,16,17].

Alemtuzumab targets mainly cells of the adaptive immune system, predominantly CD8+- and CD4+-T-cells. T-cells are mostly involved in an effective T-cell mediated bacterial clearance after infection with pathogens. It has been shown, however, that blood dendritic cells as part of the innate immune system are also depleted by alemtuzumab [18]. Dendritic cells (DCs) provide the physical link between the innate and adaptive immune system and play a crucial role in host defense against invading bacterial pathogens. Listeria monocytogenes is phagocytosed by DCs by a serum-dependent mechanism and the pattern of secreted cytokines induced by Listeria monocytogenes is dominated by interleukin-12 and -18, capable of initiating a Th-1 response [19]. Listeria meningitis induced by alemtuzumab may therefore be facilitated by immune cell depletion in the adaptive as well as the innate immune system, possibly by an outburst of a pre-existing, clinically silent and CD8 T-cell controlled infection with Listeria monocytogenes. In the two cases reported here, a latent Listeria infection must be presumed, since clinical symptoms occurred briefly after the first infusions.

We recommend that patients undergoing alemtuzumab treatment should avoid potentially contaminated animal and herbal food (e.g., raw milk products, sliced mushrooms, and smoked salmon) before and during alemtuzumab treatment. The duration of a diet sparing those products, however, is uncertain; currently there is no clear rationale to recommend a definite time interval.

In conclusion, physicians and patients should be aware of this serious, but treatable complication. In immunosuppressed patients with fever and elevated inflammatory parameters, even in the absence of meningism and headache in the beginning, Listeria meningitis should be considered.
